# Ripening Changes of the Chemical Composition, Proteolysis, and Lipolysis of a Hair Sheep Milk Mexican Manchego-Style Cheese: Effect of Nano-Emulsified Curcumin

**DOI:** 10.3390/foods10071579

**Published:** 2021-07-07

**Authors:** Mariam Sardiñas-Valdés, Hugo Sergio García-Galindo, Alfonso Juventino Chay-Canul, José Rodolfo Velázquez-Martínez, Josafat Alberto Hernández-Becerra, Angélica Alejandra Ochoa-Flores

**Affiliations:** 1 División Académica de Ciencias Agropecuarias, Universidad Juárez Autónoma de Tabasco, Carretera Villahermosa-Teapa, km 25, 86280 Villahermosa, Tabasco, Mexico; msvaldes92@gmail.com (M.S.-V.); aljuch@hotmail.com (A.J.C.-C.); jrodolfovelazquez@gmail.com (J.R.V.-M.); 2 Unidad de Investigación y Desarrollo de Alimentos, Instituto Tecnológico de Veracruz, M.A. de Quevedo # 2779, Col. Formando Hogar, 91897 Veracruz, Veracruz, Mexico; hugogg@gmail.com; 3 División de Procesos Industriales, Universidad Tecnológica de Tabasco, Carretera Villahermosa-Teapa Km 14.6, 86280 Villahermosa, Tabasco, Mexico

**Keywords:** manchego cheese, Pelibuey ewes, curcumin, ripening, proteolysis, lipolysis

## Abstract

The influence of nano-emulsified curcumin (NEC) added to the hair sheep milk, prior to cheese-making, on the chemical composition, lipolysis, and proteolysis of manchego-style cheeses were evaluated throughout 80 days of ripening. The addition of NEC to the milk resulted in cheeses with the same moisture content (42.23%), total protein (23.16%), and water activity (0.969) (*p* > 0.05). However, it increased the fat and ash levels from 26.82% and 3.64% in B 10 ppm to 30.08% and 3.85% in C 10 ppm, respectively, at the end of the ripening (*p* < 0.05). The total phenolic content and antioxidant activity of experimental cheeses increased during ripening, and the fatty acid groups showed significant changes occurred to a greater extent in the first days of ripening (*p* < 0.05). The lipolysis increased consistently in all cheeses until day 40 of ripening, to decrease at the end, while proteolysis increased during all ripening time in all samples (*p* < 0.05); the addition of NEC did not alter the primary proteolysis of manchego-style cheeses, but it modified secondary proteolysis and lipolysis (*p* < 0.05). Principal component analysis was useful for discriminating cheeses according to their chemical composition and classified into four groups according to their ripening time. This research highlights the potential of CNE to fortify dairy foods to enhance their functionality.

## 1. Introduction

In the last few decades, food products with healthy functionalities have gained more importance among consumers [1]. This has motivated the food industries to develop food products with health benefits. These food products are called functional foods, similar in appearance to conventional foods but beyond their basic nutritional functions, have demonstrated physiological benefits and can reduce the risk of chronic disease [2]. Polyphenols, flavonoids, carotenoids, terpenoids, vitamins, and fibers are some bioactive compounds used to fortify food products to enhance their functionality [3,4].

Evidence indicates that polyphenols have positive effects on human health by their biological activities, such as anti-inflammatory, antioxidant, and antimicrobial activities [3,5]. Moreover, some studies have evidenced that the polyphenol ingestion of more than 1 g/day is correlated with diminished onset and progress of chronic diseases related to oxidative stress, such as type 2 diabetes mellitus and several forms of cancer [6,7].

Curcumin, an oil-soluble polyphenolic compound with multiple functional properties, such as antioxidant, anti-inflammatory, antimutagenic, anticancer, antibacterial, antiviral, and antifungal activities, is a natural compound extracted from the rhizome of *Curcuma longa* [8]. It is not toxic even at high doses, so it exhibits the potential to be used in the food industry as coloring, flavoring, and preservative agents [9]. In addition, due to its powerful antioxidant activity, curcumin exhibits potential for use in the food industry to improve oxidative stability and extend the shelf-life of food products [10]. However, its low solubility in water, chemical instability, and poor oral bioavailability strongly limit its application in the food industry and others [11].

The use of promising novel formulations as a carrier is a feasible way to improve the bioavailability of bioactive compounds and achieve desired functionality [12]. Nano-emulsified curcumin (NEC) formulated with lecithin, medium-chain oil, glycerol, and water have demonstrated high physical stability, optical clarity, and greater bioavailability than curcumin in a coarse aqueous suspension [13]. Thus, NEC could be incorporated into a wide range of food products to extend its shelf-life and enhance its beneficial health effects.

Cheese is one of the most consumed foods worldwide and, manchego-style cheese is one of the most consumed ripened, semi-hard, pressed cheeses in Mexico [14]. It has been traditionally made exclusively from pasteurized whole cows’ milk and ripened for 14 to 30 days [15], although the original Spanish manchego cheese is made from sheep’s milk and usually commercialized after a ripening period ranging from three to eight months [16]. This milk is widely used in ripened cheeses production in Europe and some countries from Asia due to its high contents of protein, fat, and total solids; these cheeses are appreciated by consumers because of their delicate and unique aromas and flavors [17].

Milk from hair sheep (Pelibuey), raised under tropical conditions, was recently used successfully to manufacture Mexican Manchego-style cheese with suitable acceptability from consumers [18,19]. Additionally, the incorporation of NEC to the Pelibuey sheep milk used to make a manchego-style cheese matured for 60 days, not modified its flavor, texture, and overall acceptability, according to surveyed panelists [20]. However, it is required to analyze the changes that occurred during the ripening of manchego-style cheese added with NEC. Given this background, the objective of this study was to determine the chemical composition changes, proteolysis, and lipolysis during the ripening of manchego-style cheeses produced from hair sheep milk added with NEC.

## 2. Materials and Methods

### 2.1. Milk and NEC

Milk was taken from a herd of Pelibuey sheep established in the ranch “El Rodeo”, located at 17° 84′ N, 92° 81′ W; 10 masl (Tabasco, Mexico). The composition of milk was as follows: dry matter, 17.14% ± 0.19%; protein content, 5.66% ± 0.07%; fat content, 5.41% ± 0.25%; lactose content, 4.50% ± 0.07%; ash content, 1.06% ± 0.05%; acidity, 0.22% ± 0.001% of lactic acid.

NEC, formulated with 10% soy lecithin (*w*/*w*), 42.5% glycerol (*w*/*w*), 42.5% deionized distilled water (*w*/*w*), 5% medium-chain oil (*w*/*w*), and C (2.5 mg per g), prepared by the thin-film hydration-emulsification ultrasonication method [13], exhibited the following properties: droplet size, 59.98 ± 1.15 nm; polydispersity index, 0.35 ± 0.02; zeta potential, −6.88 ± 1.03 mV; C concentration, 2.50 ± 0.09 mg per g of nanoemulsion; C entrapment efficiency, 100.43% ± 0.54%.

### 2.2. Manufacturing of Mexican Manchego-Style Cheese

Mexican manchego-style cheeses were manufactured in the Laboratory of Dairy Products Technology at the Academic Division of Agricultural Sciences of the Universidad Juárez Autónoma de Tabasco. A total of 80 L of raw Pelibuey sheep’s milk was pasteurized at 63 °C for 30 min and divided into four 20 L lots. After cooling at 37 °C, the lots were subjected to different treatments: addition of bixina in solution at 10 ppm in the milk (control cheese B), the addition of NEC at 5 ppm of C in the milk (C 5 ppm), the addition of NEC at 7.5 ppm of C in the milk (C 7.5 ppm), the addition of NEC at 10 ppm of C in the milk (C 10 ppm), and four cheese variants were made according to the same procedure. A total of 0.02% (*w*/*v*) of CaCL2 and 0.002% (*w*/*v*) of lyophilized lactic acid starter culture (Bioprox M127) were added, stirred for 10 min, and allowed to stand for 30 min. Rennet of microbial origin (15 mL/100 L) from Cuamex was added. After 45 min of rennet coagulation, the curd was cut into cubes of 1 cm^3^, slowly stirred for 1 min, and left to rest for 15 min. The temperature was raised to 40 °C for 30 min for scalding and contraction of the curd. The whey was removed, and the curd molded and pressed for 18 h. The cheeses were salted by rubbing with NaCl (25 g/kg) and ripened for 80 days at a temperature of 10 °C. Each lot consisted of five cheese blocks, and each block contained approximately 500 g of cheese. Cheeses at 0, 20, 40, 60, and 80 days of ripening were taken from each lot. The cheese blocks were finely grated and kept in airtight containers at 40 °C until they were analyzed.

### 2.3. Chemical Composition of Cheeses

Moisture content, total protein, fat, and ash of the control cheese and cheeses added with NEC were determined by the methods described in AOAC [21]. Moisture content was determined by the rapid screening method in a forced-draft oven at 130 ± 1 °C (Method 948.12). Nitrogen content was determined by the macro-Kjeldahl method, and total protein content was calculated as nitrogen content multiplied by 6.38 (Method 991.20). Fat content was determined by the modified Mojonnier ether extraction method (Method 933.05). Ash content was determined by the gravimetric method in a furnace at 550 °C (Method 935.42). Water activity (aw) was measured using an Aqualab instrument at 25.5 °C. The samples were analyzed in triplicate, and data were expressed as mean ± standard deviation.

### 2.4. Total Phenolic Content (TPC) and Antioxidant Activity (AA) in Cheeses

Extraction of the cheeses to determine the TPC and AA was carried out according to the methodology described by Rashidinejad et al. [22] with some modifications. Briefly, 10 g of grated cheese were weighed, and 50 mL of 95% methanol with 1% HCl were added. The mixture was homogenized at 2000 rpm for 1 min, stirred at 200 rpm for 30 min at 50 °C, and centrifuged at 4500 rpm for 10 min to recover the supernatant. The TPC was determined by the Folin–Ciocalteu method, according to Singleton et al. [23]. A total of 250 μL of Folin–Ciocalteu reagent and 2.9 mL of DDW were added to 100 μL of the extract. The mixture was allowed to stand for 8 min, then 750 μL of a 20% sodium carbonate solution and 950 μL of DDW were added. The absorbance was measured at 760 nm, and TPC expressed as mg gallic acid equivalents (GAE) per 100 g of cheese.

The AA evaluated by the DPPH method was determined according to Brand-Williams et al. [24]. A total of 4.875 mL of DPPH reagent was added to 125 µL of the extract, and the mixture was left standing at room temperature and protected from light for one hour. Then, its absorbance was determined at 517 nm, and its AA was expressed as mmol Trolox equivalents (TE) per g of cheese. The AA evaluation through the FRAP assay was performed according to Benzie and Strain [25]; 150 μL of the extract were added to 4500 μL of the FRAP reagent and 450 μL of DDW, the mixture was left standing at room temperature and protected from light for one hour. The absorbance was measured at 593 nm, and AA was expressed in mmol TE per g of cheese.

### 2.5. Color in Manchego-Style Cheeses

The color measurement in units of L*, a*, and b* of the CIELAB color scale was performed for each cheese sample at three different sites on its surface, using a colorimeter CM-5 Konica Minolta. All analytical determinations were performed in triplicate (*n* = 3). Data were expressed as mean ± standard deviation.

### 2.6. Fatty Acid Composition of Cheeses

Cheeses fatty acid composition was determined by gas chromatography (GC) after fat extracting by the methodology of Bligh and Dyer [26], with slight modifications. Briefly, 10 g of grated sample was homogenized with 30 mL of chloroform:methanol (1:2 *v*/*v*) and 2 mL of deionized distilled water (DDW), then 10 mL of chloroform were added to homogenize again for 30 s. A total of 10 mL of DDW were added, and the mixture was homogenized once more for 30 s. The homogenate was filtered and transferred to a separatory funnel, the lower phase was drained, and the rotary evaporated. The extracted fat was derivatized with 0.25 M sodium methoxide in methanol-diethyl ether (1:1 *v*/*v*) to obtain the fatty acid methyl esters [27]. Glyceryl triundecanoate was used as an internal standard. Fatty acid methyl esters were quantified in a Perkin Elmer model AutoSystem XL gas chromatograph, fitted with a split-splitless injector, flame ionization detector (FID), and Perkin Elmer Elite Series PE-225 (30 m × 0.25 mm × 0.25 µm) capillary column. The temperature program consisted of an initial temperature of 50 °C followed by heating to 195 °C at 20 °C/min. This temperature was increased until 205 °C at 3 °C/min, and then increased by 7 °C/min until the final column temperature of 220 °C, maintained for 24 min. Injector and FID temperatures were set at 205 and 250 °C, respectively. The samples were analyzed in triplicate, and data were expressed as mean ± standard deviation.

### 2.7. Lipolysis

The lipolysis in the cheese samples was measured in terms of free fatty acids (FFAs) content, at meq of KOH per 100 g of fat, according to the method described by Deeth and Fitz-Gerald [28]. A total of 3 g of grated cheese were mixed with 5 mL of DDW and 10 mL of extraction mixture (isopropanol: petroleum ether: 4N sulfuric acid, 4:10:1). The mixture was homogenized and transferred to a stoppered test tube. A total of 6 mL of petroleum ether and 4 mL of DDW were added, and the tube was placed in a sonicator bath at 40 °C for 10 min. Then, the two layers were allowed to settle (60 min). The upper layer was withdrawn and transferred to a small flask. Finally, the solution is titrated with 0.02 N methanolic KOH solution.

### 2.8. Proteolysis

The extent of proteolysis of the manchego-style cheeses during ripening was monitored by measuring the levels of soluble nitrogen (SN) fractions, i.e., SN at pH 4.6 (pH 4.6-SN), SN in trichloroacetic acid (TCA-SN), and SN in phosphotungstic acid (PTA-SN), and prepared according to Gripon et al. [29], with slight modifications. The nitrogen content in each fraction was determined by the macro-Kjeldahl method (AOAC, 2005) and expressed as the percentage of total nitrogen (TN).

SN-pH 4.6. A total of 10 g of grated cheese was homogenized with 40 mL of a 0.5 M sodium citrate solution of pH 7.0 and kept at 40 °C for 30 min, then DDW was added to complete the volume to approximately 90 mL. A total of 1 N hydrochloric acid was added dropwise in order to lower the pH of the solution to a value of 4.6. After gentle stirring for 20 min at room temperature, the mixture was centrifuged at 4500 rpm for 10 min, filtered through Whatman No. 40 paper, and the volume adjusted to 200 mL with DDW. An aliquot of 10 mL of this solution was used for the nitrogen quantification.

TCA-SN. To 16 mL of the solution obtained to the SN-pH 4.6 described above, 4 mL of a solution of 60% TCA was added. After 1 h at room temperature, the mixture was centrifuged at 4500 rpm for 10 min and filtered through Whatman No. 40 paper. The entire soluble fraction was used for nitrogen quantification.

PTA-SN. A total of 10 mL of the filtrate of SN-pH 4.6, 5 mL of a solution of 10% PTA, and 5 mL of a 25% sulfuric acid solution were added. After 24 h of contact at room temperature, the mixture was centrifuged at 4500 rpm for 10 min and filtered through Whatman No. 40 paper. The fraction soluble was used for nitrogen quantification.

### 2.9. Data Analysis

Statistical analysis was performed using Statistica (version 6.0, StatSoft Inc., Tulsa, OK, USA). The statistical analysis, two-factor factorial ANOVA, was performed to establish the significance of two parameters, the addition of NEC to the milk and time of ripening, on the cheese chemical composition, total phenolic content and antioxidant activity, color, fatty acid composition, lipolysis, and proteolysis, the level of statistical significance being set at *p* < 0.05.

A principal component analysis (PCA) and a hierarchical cluster analysis (HCA) were developed considering the variables of moisture, fat, total protein, ash, a_w_, TPC, AA by DPPH and FRAP, CIE L*, a*, and b*, FFAs, pH4.6-SN/TN, TCA-SN/TN, and PTA-SN/TN, for all of the treatments evaluated (B 10 ppm, C 5 ppm, C 10 ppm, and C 7.5 ppm). The PCA analysis was performed considering the correlation matrix and the HCA analysis between-groups linkage cluster method. The multivariate analyses were performed using Minitab^®^ Statistical Software release 19.2020.2.0 (Minitab Inc., State College, PA, USA).

## 3. Results and Discussion

### 3.1. Chemical Composition of Cheeses

The chemical composition of the manchego-style cheeses at different ripening times is shown in Table 1. Its composition was similar to that reported for manchego cheese by other authors [14,30,31]. The addition of NEC to the milk at concentrations of 5 and 7.5 ppm did not statistically influence significantly (*p* > 0.05) on the content of moisture, total protein, and ash of the cheeses. The differences in the values for these chemical parameters were only significant (*p* < 0.05) for cheeses added with the highest concentration of NEC tested (10 ppm). The fat content showed statistically significant differences at 10 ppm of added curcumin, compared to the control cheese, and also at 7.5 ppm of curcumin addition, but only at 60 days of storage. Therefore, it possible that a higher concentration of NEC could have a more significant effect on the composition of cheese than observed herein. The addition of NEC did not statistically influence significantly (*p* > 0.05) the parameter of water activity. The ripening time statistically significantly influenced all of the investigated parameters (moisture, fat, total protein, ash, and water activity).

At the beginning of the ripening time, no differences in the basic chemical composition of cheese samples were observed. It is explained by the fact that all the cheeses were made with the same batch of milk and according to the same procedure. However, some authors have reported that the addition of polyphenolic bioactive compounds to milk promotes the contraction of the cheese matrix and the expulsion of whey, reducing the quantity of entrapped water in the protein [32,33]. In this work, the moisture content of the cheeses made with milk added with NEC decreased slightly compared to the control cheese, although these differences were not statistically significant (*p* > 0.05), observing a higher ash content in cheeses with lower moisture content.

During maturation, the cheeses’ moisture content and water activity decrease significantly (*p* < 0.05), while their total protein and ash content also increases significantly. According to Lesic et al. [34], the moisture loss can be caused by lactic acid production during ripening, the synaeresis taking place in the cheese, and/or the reduced casein hydration as the pH reaches its iso-electric point. The total protein and ash content were related to the moisture content reached during ripening.

In relation to the total fat content in the cheeses decreased significantly from day zero to day 40, while from day 40 to day 80, it increased significantly. According to Atasoy and Türkoglu [35], it can be due to the hydrolysis of triglycerides in milk fat, which is related to the increase in free fatty acids in the cheese mass during its maturation. Moreover, Virto et al. [36] and Cabezas et al. [30] reported that the major changes observed in its lipids content occurred during the first 30 days of ripening for all the cheese samples studied. The increase in total fat content at the end of ripening was also related to the moisture content reached during maturation.

### 3.2. Total Phenolic Content (TPC) and Antioxidant Activity (AA) in Cheeses

Table 2 shows the TPC and AA of the cheese samples at different ripening times. A statistically significant influence of both the addition of NEC to the milk (*p* < 0.05) and the ripening time (*p* < 0.05) was observed for the TPC and AA of the cheeses. Higher values of TPC and AA were found for the cheeses added with NEC compared to the control cheese due to the addition of curcumin, a natural polyphenolic compound with potent antioxidant activity [37]. Rashidinejad et al. [38] also found that the fortification of cheeses with different products rich in polyphenols significantly increased their TPC and AA.

On the other hand, the TPC and AA increased during the ripening time, both for the cheeses added with NEC and the control cheese. At the beginning of the ripening time, all tested samples exhibited the average TPC value of 118.26 mg GAE/100 g, the DPPH value was 10.82 mmol TE/g, and the FRAP value was 3.13 mmol TE/g. These values increased significantly during ripening (*p* < 0.05) and, at the end of the ripening time (80 d), the average values reached 291.40 mg GAE/100 g to TPC, 12.49 mmol TE/g in the DPPH assay, and 4.08 mmol TE/g in the FRAP assay, increases of about 146%, 15%, and 30% over the initial value, respectively. These results are consistent with those reported by other authors such as Batool et al. [39], Khan et al. [40], and Perna et al. [41], who indicate that the increase in AA is closely related to the increase in the concentration of antioxidant compounds generated during ripening, mainly biopeptides, with beneficial health effects.

According to Rashidinejad et al. [22], the progressive proteolysis of proteins by the presence of chymosin residual, plasmin, and starter and nonstarter bacterial proteases and peptidases produce water-soluble peptides and free amino acids with antioxidant capacity. González-Martín et al. [42] reported that the total content of water-soluble peptides in cheeses of cow, sheep, and goat increased throughout ripening, being more extensive in the first month of maturity. This also was reported by Gupta et al. [43], who indicated that the antioxidant activity is higher in cheeses with a higher degree of early proteolysis.

**Table 2:** Total phenolic content and antioxidant activity (evaluated by the FRAP and DPPH assays) in the manchego-style cheeses at different ripening times.

Cheese Samples	Ripening Time (days)	Total Phenolic Content	Antioxidant Activity by DPPH	Antioxidant Activity by FRAP
B 10 ppm	0	104.62 ± 4.37 ^a1^	10.24 ± 0.08 ^a1^	2.67 ± 0.18 ^a1^
	20	171.95 ± 7.71 ^b1^	10.79 ± 0.23 ^ab1^	2.78 ± 0.21 ^a1^
	40	206.30 ± 3.33 ^c1^	10.95 ± 0.17 ^b1^	3.33 ± 0.19 ^b1^
	60	213.49 ± 3.56 ^c1^	11.66 ± 0.81 ^c1^	3.45 ± 0.03 ^bc1^
	80	251.53 ± 13.81 ^d1^	10.62 ± 0.42 ^ab1^	3.61 ± 0.12 ^c1^
C 5 ppm	0	111.96 ± 2.98 ^a12^	10.57 ± 0.37 ^a12^	2.98 ± 0.05 ^a2^
	20	183.62 ± 3.31 ^b2^	11.30 ± 0.10 ^b12^	3.44 ± 0.10 ^b2^
	40	241.84 ± 3.85 ^c2^	11.48 ± 0.14 ^b12^	3.58 ± 0.08 ^bc12^
	60	245.65 ± 8.81 ^c2^	11.90 ± 0.39 ^b1^	3.74 ± 0.30 ^c2^
	80	286.04 ± 14.66 ^d2^	11.28 ± 0.27 ^b2^	3.82 ± 0.12 ^c2^
C 7.5 ppm	0	119.23 ± 0.06 ^a2^	11.04 ± 0.27 ^a23^	3.27 ± 0.01 ^a3^
	20	187.73 ± 4.58 ^b23^	11.51 ± 0.04 ^ab2^	3.63 ± 0.05 ^b23^
	40	253.29 ± 7.41 ^c2^	11.91 ± 0.17 ^b23^	3.90 ± 0.11 ^c2^
	60	280.90 ± 3.13 ^d3^	12.66 ± 0.47 ^c2^	4.27 ± 0.09 ^d3^
	80	309.68 ± 5.44 ^e3^	11.75 ± 0.14 ^b23^	4.27 ± 0.03 ^d3^
C 10 ppm	0	137.22 ± 7.41 ^a3^	11.44 ± 0.17 ^a3^	3.59 ± 0.12 ^a4^
	20	197.20 ± 6.00 ^b3^	12.22 ± 0.41 ^b3^	3.89 ± 0.24 ^b3^
	40	267.24 ± 1.02 ^c3^	12.41 ± 0.20 ^b3^	4.55 ± 0.10 ^c3^
	60	307.91 ± 4.41 ^d4^	13.73 ± 0.64 ^c3^	4.73 ± 0.10 ^c4^
	80	318.34 ± 8.03 ^d3^	12.30 ± 0.54 ^b3^	4.64 ± 0.11 ^c4^

Total phenolic content values are expressed as mg gallic acid equivalents (GAE) per 100 g of cheese, and antioxidant activity values are expressed as mmol Trolox equivalents (TE) per g of cheese. Results are expressed as mean (*n* = 3) ± standard deviation. ^a–e^ Means in each column with different letters were significantly affected by ripening time (*p* < 0.05); ^1–4^ means with different numbers were significantly different between cheese samples at a similar ripening time (*p* < 0.05).

On the other hand, the TPC and AA increased during the ripening time, both for the cheeses added with NEC and the control cheese. At the beginning of the ripening time, all tested samples exhibited the average TPC value of 118.26 mg GAE/100 g, the DPPH value was 10.82 mmol TE/g, and the FRAP value was 3.13 mmol TE/g. These values increased significantly during ripening (*p* < 0.05) and, at the end of the ripening time (80 d), the average values reached 291.40 mg GAE/100 g to TPC, 12.49 mmol TE/g in the DPPH assay, and 4.08 mmol TE/g in the FRAP assay, increases of about 146%, 15%, and 30% over the initial value, respectively. These results are consistent with those reported by other authors as Batool et al. [39], Khan et al. [40], and Perna et al. [41], who indicate that the increase in AA is closely related to the increase in the concentration of antioxidant compounds generated during ripening, mainly biopeptides, with beneficial health effects.

According to Rashidinejad et al. [22], the progressive proteolysis of proteins by the presence of chymosin residual, plasmin and starter and nonstarter bacterial proteases and peptidases produce water-soluble peptides and free amino acids with antioxidant capacity. González-Martín et al. [42] reported that the total content of water-soluble peptides in cheeses of cow, sheep, and goat increased throughout ripening, being more extensive in the first month of maturity. This also was reported by Gupta et al. [43], who indicated that the antioxidant activity is higher in cheeses with a higher degree of early proteolysis.

### 3.3. Color in Manchego-Style Cheeses

Color is an important criterion for evaluating the quality of cheeses and is often the primary consideration for consumers when making their purchase decision [44]. The color measurements of the manchego-style cheeses at the different ripening periods are shown in Table 3. These scores indicate a significant difference between the different samples of cheeses and between the different ripening times (*p* < 0.05). The average L* and a* values were lower in curcumin-enriched cheeses than the control cheese, while the b* value was higher in the cheeses with NEC.

There is also a close relationship between the amount of curcumin added to the milk and the values of L*, a*, and b* obtained in the cheese samples. When the amount of curcumin is increased, L* and b* also increase, while the values of a* decrease. Brightness (L* value) and yellowness (b* value) increase with increasing curcumin content since curcumin is a bright yellow compound. Tarakci et al. [45] similarly reported that in a Turkish cheese, the value in a* decreased as the concentration of additive increased, while Giroux et al. [32] reported an increase in the b* value as the concentration of additive increased, which accentuates the yellow color of the cheese.

**Table 3:** Color, in units of L*, a*, and b * in the manchego-style cheeses at different ripening times.

Cheese Samples	Ripening Time (days)	L*	a*	b*
B 10 ppm	0	78.27 ± 0.85 ^a^	− 2.27 ± 0.15 ^a1^	6.00 ± 0.75 ^a1^
	20	77.67 ± 0.45 ^a^	0.23 ± 0.49 ^b1^	9.93 ± 0.90 ^b1^
	40	77.90 ± 0.70 ^a1^	0.67 ± 0.12 ^b1^	9.73 ± 1.65 ^b1^
	60	77.13 ± 1.51 ^ab1^	0.57 ± 0.67 ^b1^	11.57 ± 1.66 ^c1^
	80	75.33 ± 1.27 ^b12^	0.43 ± 0.29 ^b1^	11.70 ± 2.29 ^c1^
C 5 ppm	0	76.57 ± 1.50 ^a^	− 3.17 ± 0.15 ^a1^	10.53 ± 0.98 ^a2^
	20	76.43 ± 1.50 ^a^	− 3.20 ± 0.69 ^a2^	13.57 ± 0.99 ^b2^
	40	74.80 ± 0.96 ^ab2^	− 1.47 ± 0.83 ^b2^	18.90 ± 0.82 ^c3^
	60	75.03 ± 0.49 ^ab12^	− 0.90 ± 0.78 ^bc23^	18.07 ± 2.12 ^c2^
	80	72.97 ± 0.35 ^b3^	− 0.27 ± 0.50 ^c1^	17.70 ± 2.21 ^c2^
C 7.5 ppm	0	76.57 ± 0.25 ^ab^	− 4.93 ± 0.15 ^a2^	12.37 ± 1.05 ^a23^
	20	78.27 ± 1.31 ^a^	− 4.87 ± 1.12 ^a3^	15.20 ± 1.11 ^b23^
	40	74.80 ± 1.23 ^bc2^	− 1.40 ± 0.72 ^b2^	19.00 ± 1.84 ^c3^
	60	74.03 ± 1.04 ^c2^	− 0.73 ± 0.76 ^b2^	18.80 ± 1.77 ^c2^
	80	74.77 ± 1.40 ^bc23^	− 0.43 ± 0.55 ^b1^	22.60 ± 2.51 ^d3^
C 10 ppm	0	77.63 ± 0.78	− 5.17 ± 0.21 ^a2^	14.17 ± 0.75 ^a3^
	20	76.73 ± 2.12	− 5.80 ± 0.46 ^a3^	18.43 ± 1.95 ^b3^
	40	77.37 ± 1.02 ^1^	− 2.13 ± 0.47 ^b2^	13.80 ± 0.95 ^a2^
	60	76.50 ± 0.36 ^1^	− 1.90 ± 0.78 ^b3^	20.30 ± 0.90 ^b2^
	80	77.27 ± 1.12 ^1^	− 2.10 ± 0.70 ^b2^	24.10 ± 3.73 ^c3^

Results are expressed as mean (*n* = 3) ± standard deviation. ^a–d^ Means in each column with different letters were significantly affected by ripening time (*p* < 0.05); ^1–4^ means with different numbers were significantly different between cheese samples at a similar ripening time (*p* < 0.05). L* brightness, a* red/green coordinates, b* yellow/blue coordinates.

On the other hand, the brightness of the manchego-style cheeses decreased significantly during the ripening (*p* < 0.05) while yellowness and redness increased significantly (*p* < 0.05), see Table 3. At the beginning of the ripening time, the tested samples exhibited an average L* value of 77.26, which decreased during the storage period until reaching, at the end of the ripening (80 d), an average value of 75.08, indicating cheeses with lower brightness. The above was also observed in the studies conducted by other authors as Pinho et al. [46] in Terrincho cheese, El-Nimr et al. [47] in Gouda cheese, Tarakci et al. [45] in herby pickled cheese, and Lee et al. [48] in Appenzeller cheese. Ibáñez et al. [49] reported that the increase in proteolysis and the reduction in the proportion of insoluble calcium could reduce L* values.

Redness (a* value) in the cheese samples increased with ripening time. In the beginning, with an average value of −3.88, it reached at the end of period (80 d) an average value of −0.59; this is in agreement with the results of Tarakci et al. [45] and El-Nimr et al. [47] in herby pickled and Egyptian Gouda cheeses, respectively. Juric et al. [50] and Kristensen et al. [51] also studied the color changes during storage in slices of semi-hard cheeses packed in modified atmospheres, reporting increases in the redness of the cheeses during the ripening time.

Yellowness (b* value) showed an increase during the ripening of the cheeses. In the beginning, the tested samples exhibited an average value of 10.77, which increased until reaching an average value of 19.03, at the 80 d of ripening. These results agree with those of other authors such as Lee et al. [48], Pinho et al. [46], Pillonel et al. [52], and Rohm and Jaros [53]. However, Tarakci et al. [45] and Juric et al. [48] reported a decrease in b* value during ripening in herby pickled cheese and semi-hard cheeses, respectively. In this sense, Juric et al. [50] indicated that samples stored in the dark were significantly more yellow than samples exposed to light, possibly due to light-induced degradation of the carotenoids and the degradation of riboflavin, which acts as a photosensitizer. Rohm and Jaros [53] showed that the color of the cheese is affected mainly by the qualitative properties of the fat phase, such as its beta-carotene content, in addition to other factors that include the total solids content well as the changes induced by cheese maturation.

### 3.4. Fatty Acid Composition of Cheeses

**Figure 1:**
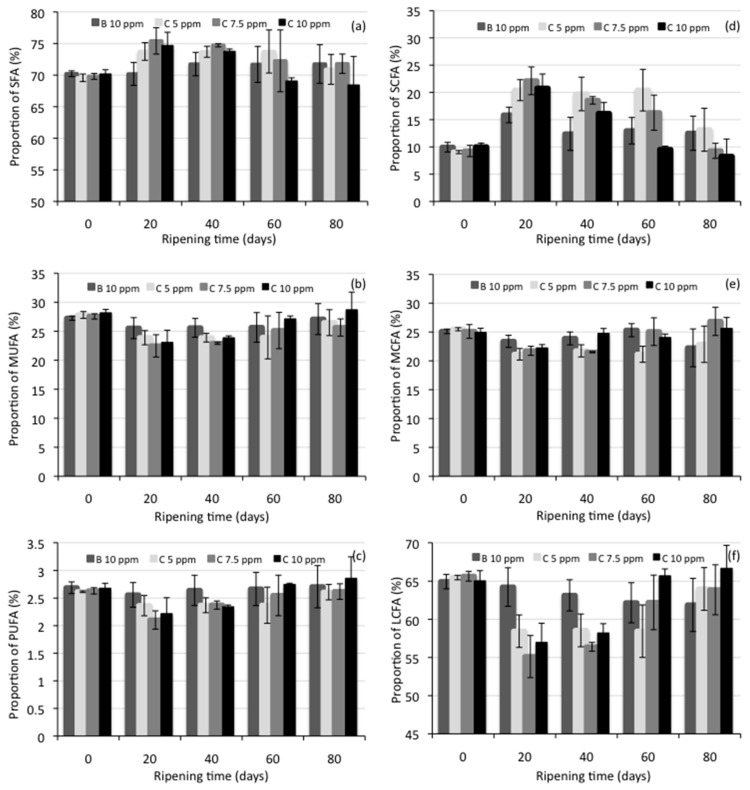
Proportion of fatty acid groups in Manchego-style cheeses at different ripening times; (**a**) saturated (SFA), (**b**) mono-unsaturated (MUFA), (**c**) polyunsaturated (PUFA), (**d**) short-chain (SCFA), (**e**) medium-chain (MCFA), and (**f**) long-chain (LCFA) fatty acids. SCFA = C4-C8; MCFA = C10–C15; LCFA = C16–C18. B = bixin, C = curcumin.

Individual fatty acids found in the manchego-style cheese on ripening day 40 are shown in Table 4, while Figure 1 shows the proportion of fatty acid groups (saturated, SFA; mono-unsaturated, MUFA; polyunsaturated, PUFA; short-chain, SCFA; medium-chain, MCFA; long-chain, LCFA) in the cheese samples at the different ripening times (Tables S1–S6 can be consulted in the Supplementary Materials to see the data from Figure 1a–f). In all samples, 21 fatty acids were identified. Statistical analysis showed significant differences (*p* < 0.05) in contents of 7 individual fatty acids (4:0, 12:0, 14:0, 14:1 *n*-5, 15:0, 15:1 *n*-7, 18:3 *n*-3) dependent on the cheese type (control cheese, added with bixin at 10 ppm, or cheeses added with NEC at 5, 7.5, or 10 ppm of C), while the influence of ripening time was found to be statistically significant (*p* < 0.05) for the content of 13 fatty acids (4:0, 6:0, 12:0, 14:0, 14:1 *n*-5, 15:0, 15:1 *n*-7, 16:0, 16:1 *n*-7, 17:0, 18:1 *n*-9, 18:2 *n*-6, 18:3 *n*-3). The analysis of variance for the proportion of fatty acid groups in the cheese samples showed a statistically significant effect (*p* < 0.05) of the cheese type in SCFA (C4-C8), MCFA (C10-C15), and LCFA (C16-C18), while the different times of ripening showed a significant difference (*p* < 0.05) in all fatty acid groups (SFA, MUFA, PUFA, SCFA, MCFA, and LCFA).

In the same way, as for the chemical composition of cheese samples, there were no significant differences in the individual fatty acid contents, or in the proportion of fatty acid groups, for all the cheese samples at the beginning of ripening. During maturation, and as it was reported by Virto et al. [36], the major changes observed in the fatty acid composition of cheese samples occurred in the first days of ripening (see Figure 1); the SCFA content in the cheeses increased significantly by day 20, compared to day zero of maturation, while MCFA and LCFA decreased.

**Table 4:** Fatty acid composition of the manchego-style cheeses on ripening day 40.

Fatty Acid	Cheese Samples			
	B 10 ppm	C 5 ppm	C 7.5 ppm	C 10 ppm
4:0	5.49 ± 2.08 ^a^	11.90 ± 2.02 ^b^	8.14 ± 2.81 ^ab^	6.63 ± 2.66 ^ab^
6:0	3.94 ± 1.67 ^a^	4.84 ± 0.51 ^a^	5.74 ± 1.12 ^a^	5.11 ± 1.56 ^a^
8:0	2.97 ± 0.53 ^a^	2.98 ± 0.28 ^a^	4.67 ± 1.48 ^a^	4.41 ± 1.19 ^a^
10:0	7.07 ± 0.68 ^a^	6.72 ± 0.75 ^a^	6.34 ± 0.43 ^a^	7.43 ± 0.68 ^a^
12:0	4.59 ± 0.30 ^a^	4.13 ± 0.46 ^ab^	3.98 ± 0.01 ^b^	4.68 ± 0.27 ^a^
14:0	10.44 ± 0.50 ^a^	9.27 ± 0.57 ^b^	9.59 ± 0.17 ^b^	10.75 ± 0.65 ^a^
14:1 *n*-9	0.21 ± 0.01 ^a^	0.20 ± 0.01 ^a^	0.21 ± 0.01 ^a^	0.22 ± 0.02 ^a^
14:1 *n*-7	0.22 ± 0.02 ^a^	0.19 ± 0.02 ^a^	0.19 ± 0.02 ^a^	0.22 ± 0.01 ^a^
14:1 *n*-5	0.40 ± 0.02 ^a^	0.36 ± 0.01 ^b^	0.36 ± 0.01 ^b^	0.42 ± 0.03 ^a^
15:0	0.75 ± 0.07 ^ab^	0.65 ± 0.05 ^a^	0.71 ± 0.02 ^a^	0.79 ± 0.04 ^b^
15:1 *n*-7	0.22 ± 0.01 ^a^	0.22 ± 0.01 ^a^	0.23 ± 0.01 ^a^	0.24 ± 0.01 ^a^
16:0	25.48 ± 1.68 ^a^	23.22 ± 1.01 ^a^	22.80 ± 2.56 ^a^	23.36 ± 2.79 ^a^
16:1 *n*-9	0.45 ± 0.02 ^a^	0.40 ± 0.03 ^a^	0.39 ± 0.06 ^a^	0.41 ± 0.07 ^a^
16:1 *n*-7	0.52 ± 0.14 ^a^	0.70 ± 0.15 ^a^	0.41 ± 0.14 ^a^	0.46 ± 0.15 ^a^
16:1 *n*-3	0.32 ± 0.01 ^a^	0.32 ± 0.01 ^a^	0.29 ± 0.04 ^a^	0.30 ± 0.06 ^a^
17:0	0.49 ± 0.03 ^a^	0.45 ± 0.03 ^a^	0.44 ± 0.05 ^a^	0.45 ± 0.09 ^a^
17:1 *n*-8	0.22 ± 0.01 ^a^	0.22 ± 0.01 ^a^	0.23 ± 0.02 ^a^	0.28 ± 0.07 ^a^
18:0	9.97 ± 0.70 ^a^	9.54 ± 0.31 ^a^	8.84 ± 0.80 ^a^	9.20 ± 0.91 ^a^
18:1 *n*-9	23.03 ± 1.81 ^a^	21.25 ± 0.66 ^a^	20.61 ± 2.78 ^a^	21.42 ± 2.86 ^a^
18:2 *n*-6	2.22 ± 0.22 ^a^	2.01 ± 0.10 ^a^	2.05 ± 0.26 ^a^	2.05 ± 0.25 ^a^
18:3 *n*-3	0.45 ± 0.05 ^a^	0.35 ± 0.05 ^ab^	0.32 ± 0.11 ^ab^	0.29 ± 0.06 ^b^

Results are expressed as % of total fatty acids as mean (*n* = 3) ± standard deviation. Means with different letters are significantly different (*p* < 0.05) between cheese samples.

The increase in the content of SCFA was found to be due solely to the increase in butyric acid content, which was also reported by Lesic et al. [34] in cow and sheep milk cheeses produced with different starter cultures and ripened for 45 days. An increase in SCFA, mainly butyric acid, was also reported by Virto et al. [36] in idiazabal cheeses made from sheep’s milk and ripened for 90 days. However, it is also observed in Figure 1 that the SCFA content in cheeses decreases at the end of maturation. The interpretation of these results can be explained by taking into account the distribution of the fatty acids within the triacylglyceride molecules.

According to Collins et al. [54], on the triacylglyceride molecules, the SCFA are located predominantly at position sn-3, and the lipases involved in cheese ripening tend to be specific for position sn-3 so that it mainly hydrolyzes SCFA. Part of these fatty acids released change into methyl ketones, lactones, secondary alcohols, and other compounds that contribute to cheese flavor.

At the end of ripening (day 80), SCFA, MCFA, and LCFA represented approximately 8%–13%, 22%–27%, and 62%–67%, respectively, of all fatty acids in all experimental cheeses. Butyric acid (4:0) was the main SCFA in all cheeses, myristic acid (14:0) was the predominant MCFA, and palmitic acid (16:0) was the main LCFA. SFA, MUFA, and PUFA presented ranges of 68%–72%, 26%–29%, and 2.6%–2.9%, respectively. Oleic acid represents 90% of the total MUFAs present in the cheeses. This fatty acid composition was similar to that reported by Atasoy and Türkoglu [35] for an urfa cheese of 90 days of maturation, a traditional Turkish cheese manufactured from raw milk ovine. Prandini et al. [55] also reported a similar fatty acid composition in 18 commercial Italian and French cheeses produced from sheep milk.

### 3.5. Lipolysis

**Figure 2:**
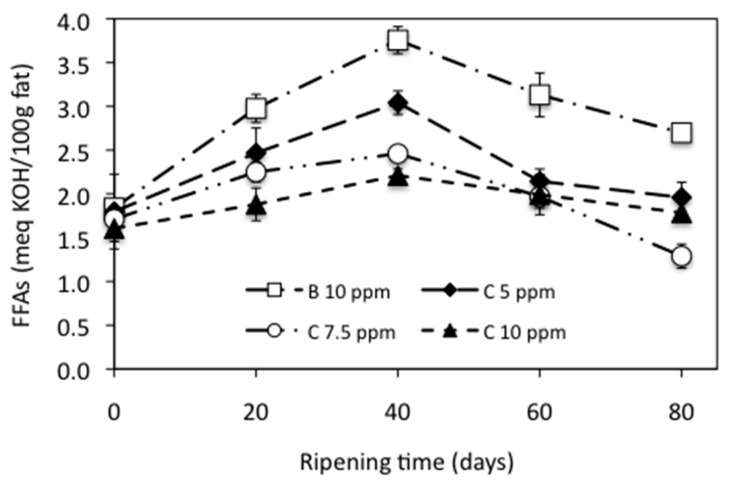
Lipolysis of manchego-style cheeses ripened at 10 °C, expressed as free fatty acids (FFAs) content. B = bixin, C = curcumin.

Fat in cheeses can be degraded b the action of lipases originating from milk, rennet, starter culture bacteria, or nonstarter bacteria, thus resulting in the liberation of free fatty acids (FFAs) during cheese ripening, which may directly contribute to cheese flavor or act as precursors of other flavor components such as aldehydes, ketones, lactones, or aliphatic and aromatic esters. Figure 2 shows the lipolysis evolution expressed as FFAs content, at meq of KOH per 100 g of fat, in the cheese samples during ripening (Table S7 can be consulted in the Supplementary Materials to see the data from Figure 2). A statistically significant influence of both the addition of NEC to the milk (*p* < 0.05) and the ripening time (*p* < 0.05) was observed for the FFAs content in the cheeses.

Lower average values for the FFAs content were found for the cheeses added with NEC (2.28, 1.94, and 1.89 meq KOH/100 g fat, to 5, 7.5, and 10 ppm of C, respectively) compared to the control cheese (2.88 meq KOH/100 g fat). These results are similar to those obtained by Kurcubic et al. [56] in a Serbian cheese added with an extract of Kitaibelia vitifolia and Khalifa and Wahdan [57] in a soft white cheese added with a blueberry extract. However, the addition of wild garlic to a Turkish cheese leads to a significant increase in the FFAs content compared to the control cheese [45]. As reported by Del Olmo et al. [58], these differences may be due to the nature of the materials used and their lipolytic activity, the characteristics of the extracts, or the cheese-making process.

Moreover, hydrolysis of triglycerides mainly depends upon the moisture content, temperature, metal ion contamination, and concentration of lipases [39]. Control cheese had a higher average moisture content (45.37%) than cheeses added with NEC (44.68%, 43.67%, and 42.89%, to 5, 7.5, and 10 ppm of C, respectively), this could be an appropriate justification for the higher content of free fatty acids.

The FFAs content of the experimental cheeses increased consistently as the ripening period progressed until day 40 (2.87 meq KOH/100 g fat), the minimum FFAs (1.74 meq KOH/100 g fat) was found at day 0, and 1.93 meq KOH/100 g fat were obtained at day 80. Between 40 and 80 days of ripening, FFAs produced by lipolysis decreased significantly. Our results were in agreement with the result of Poveda et al. [59] for manchego cheese and Partidario et al. [60] for Serra de Estrela cheese. Poveda et al. [61] also, for manchego cheese, reported a non-uniform behavior for the different FFAs with ripening time, partly due to esters formation.

Partidario et al. [60] indicated that FFAs, mainly short-chain, undergo extensive transformations such as volatilization, esterification, and bacterial catabolism after being liberated from the triglycerides. Analysis of volatile compounds showed an increase in the total concentration of ketones in the cheeses from 3 to 6 weeks of ripening; this increase corresponds to a decrease in the concentration of total FFAs and short-chain FFAs, which suggests that the production of ketones is a consequence of fatty acids oxidation [60]. Poveda et al. [59] indicated that the concentration of FFAs during the ripening decreased, possibly because the FFAs could have been hydrolyzed into other compounds, such as methyl ketones, alcohols, lactones, or aldehydes.

### 3.6. Proteolysis

**Figure 3:**
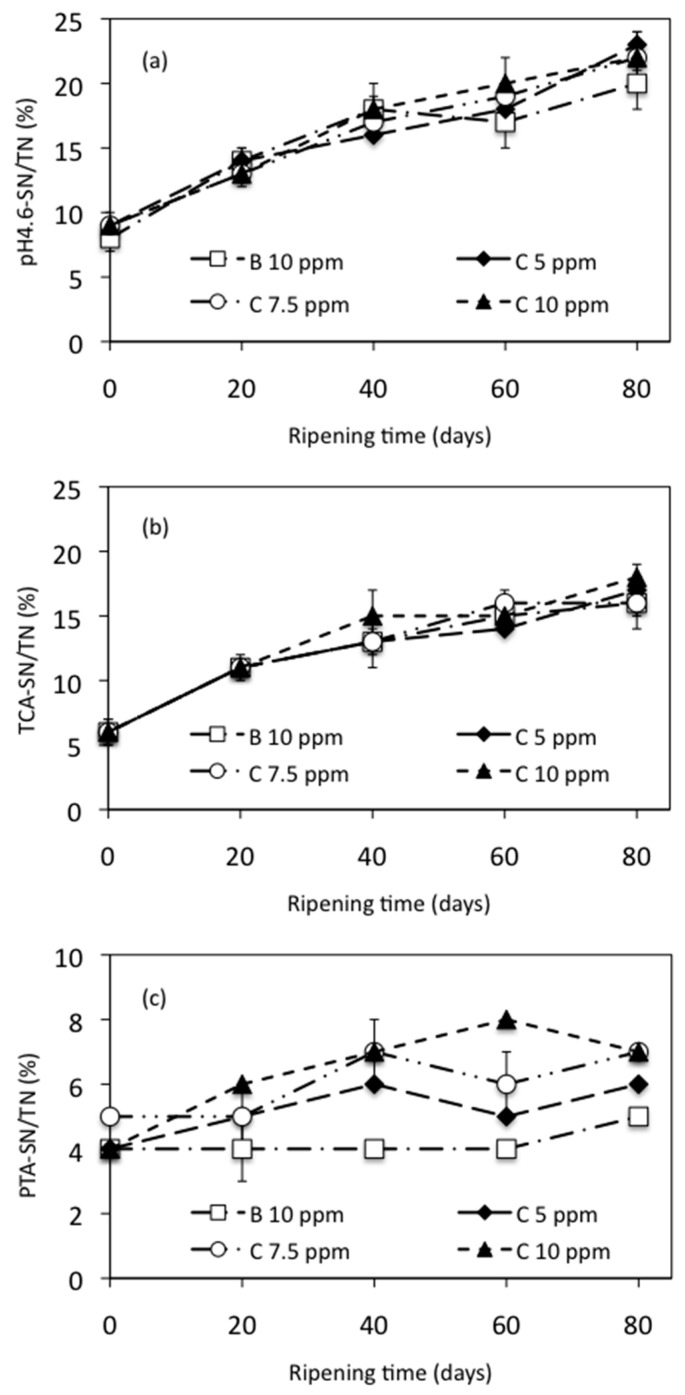
Proteolysis of manchego-style cheeses ripened at 10 °C, expressed as (**a**) soluble nitrogen (SN) at pH 4.6 (pH 4.6-SN)/total nitrogen (TN), (**b**) in trichloroacetic acid (TCA-SN)/TN and (**c**) in phosphotungstic acid (PTA-SN)/TN. B = bixin, C = curcumin.

Proteolysis is one of the most important processes during cheese ripening. During this process, some peptides and amino acids are released, constituting the SN fractions [32]. It recently has been recognized that various of these peptides may exert beneficial effects, such as the tripeptides Val-Pro-Pro and Ile-Pro-Pro, which have been isolated from different cheese varieties and have demonstrated ACE-inhibiting potential to exert a blood pressure-lowering effect in humans with mild hypertension [62]. On the other hand, the proteolysis in the cheese also results in the appearance of biogenic amines, which are not considered a severe risk in cheeses but could be helpful as indicators of freshness and hygienic quality of the raw materials and the manufacturing conditions used within cheese manufacture [63]. Figure 3 shows the proteolysis of manchego cheese ripened at 10 ºC expressed as fractions of pH 4.6-SN/TN (a), TCA-SN/TN (b), and PTA-SN/TN (c). (Tables S8–S10 can be consulted in the Supplementary Materials to see the data from Figure 3a–c).

Levels of 4.6-SN/TN, TCA-SN/TN, and PTA-SN/TN increased during ripening as a consequence of proteolysis in all trials. The levels of pH 4.6-SN/TN at 0, 20, 40, 60, and 80 days did not differ significantly (*p* > 0.05) between cheeses with and without added NEC; see Figure 3a.

These results show that no influence of NEC was detected on primary proteolysis, which is not surprising taking into account that this process is mainly catalyzed by residual chymosin and, to a lower degree, by other proteinases present in the curd such as plasmin or cell envelope proteases from the starter [64]. According to Vivar-Quintana et al. [65] and Bergamini et al. [66], as the cheese ages, more caseins and high molecular weight peptides are broken down into smaller peptides that can be water-soluble; therefore, as cheese ages, the total content of water-soluble peptides increases.

In the same way, the level of TCA-SN/TN did not show significant differences (*p* > 0.05) between control cheese and cheeses added with NEC during the time ripening, see Figure 3b. These results suggest that NEC added to cheeses did not influence the production of medium- and small-sized peptides. On the contrary, the PTA-SN/TN levels were significantly different (*p* < 0.05) between cheeses with and without added NEC either at 20, 40, 60, or 80 days of ripening (see Figure 3c). All of the experimental cheeses added with NEC showed higher levels of PTA-SN/TN than control cheese. Moreover, there is a close relationship between the amount of NEC added to the milk and the levels of PTA-SN/TN obtained in the cheese samples; when the amount of NEC is increased, the levels of PTA-SN/TN also increase.

The average value obtained for PTA-SN/TN on the control cheese was 4.28%, while on the cheeses added with NEC, the average values were 5.14%, 5.86%, and 6.48% to 5, 7.5, and 10 ppm of C, respectively. These results suggest that the addition of NEC did influence the release of very small peptides, amino acids, and other smaller nitrogenous compounds such as urea, amines, and ammonia [67,68]. Our observations agreed with those obtained by Bergamini et al. [66] and Gardiner et al. [69] to Argentino semi-hard cheese and Cheddar cheese, respectively, both manufactured with probiotic culture. These results show that the addition of NEC to the hair sheep milk alter secondary proteolysis of the cheeses, but not primary proteolysis.

### 3.7. Principal Component Analysis

**Figure 4:**
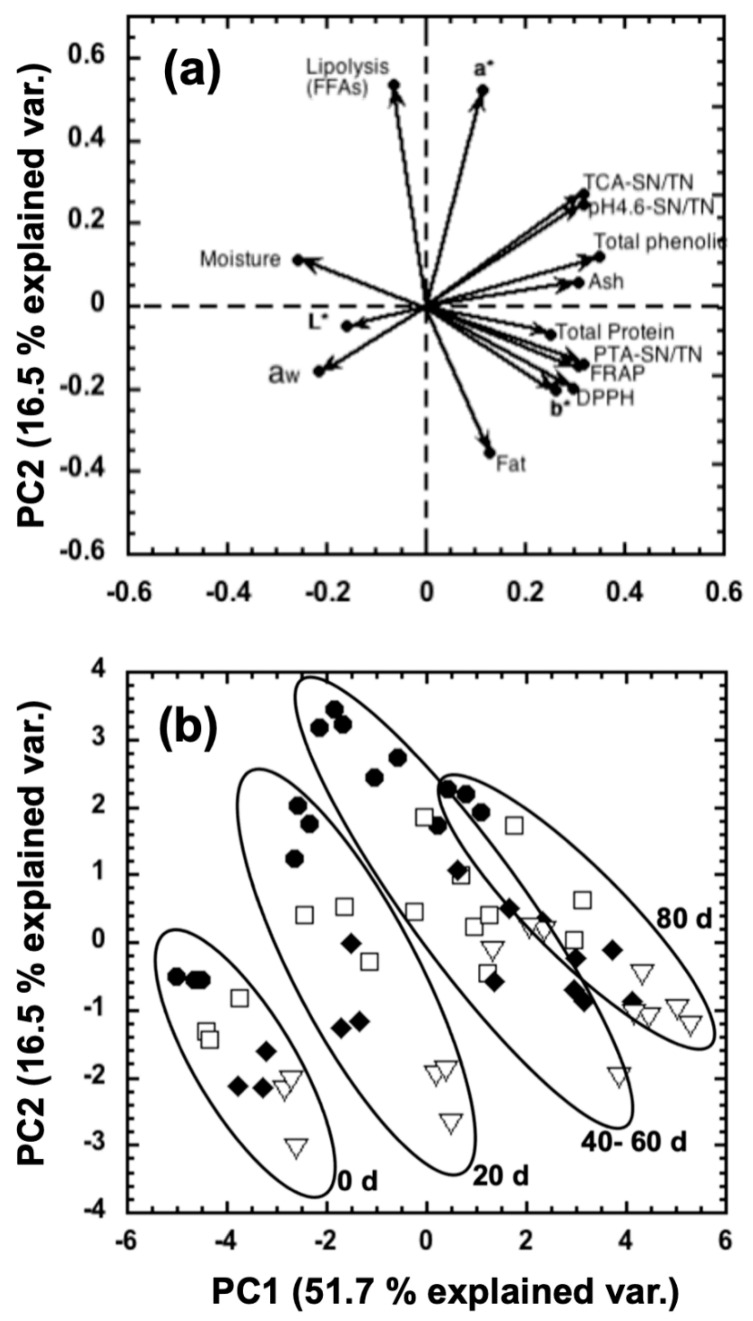
Loading plot (**a**) and score plot (**b**) obtained by principal component analysis (PCA) from different variables of manchego-style cheeses added with bixin 10 ppm (⯃), NEC 5 ppm (□), NEC 7.5 ppm (⯁), and NEC 10 ppm (▽), throughout 0, 20, 40, 60, and 80 days of ripening.

To summarize the effect of the addition of NEC to the hair sheep milk on the chemical composition, proteolysis, and lipolysis of manchego-style cheeses throughout ripening time, a principal component analysis (PCA) was applied. A bi-dimensional representation of the two principal components (Figure 4) shows that both principal components explained 68.2% of the total variance.

Figure 4a (loading plot) shows that the first principal component (PC1) was negatively correlated with the moisture, a_w_, L*, and FFAs, and positively correlated with fat, total protein, ash, TPC, AA by DPPH and FRAP, a*, b*, pH4.6-SN/TN, TCA-SN/TN, and PTA-SN/TN. On the other hand, the PC2 was positively correlated with FFAs, a*, TCA-SN/TN, pH4.6-SN/TN, TPC, moisture, and ash, and negatively correlated with fat, b*, a_w_, AA by DPPH and FRAP, PTA-SN/TN, total protein, and L*. Through the hierarchical cluster analysis (HCA), it was possible to distinguish groups of samples. In this sense, the cheeses could be grouped into four groups based on ripening time: 0, 20, 40–60, and 80 days (Figure 4b).

It is important to highlight the influence of the variables related to the proteolysis of cheeses (pH4.6-SN/TN, TCA-SN/TN, PTA-SN/TN) on PC1 and its behavior throughout ripening time (Figure 4a). Mezo-Solis et al. [18] also reported a great influence of the variables used to estimate proteolysis (pH4.6-SN/TN, 12%TCA-SN) on the PC1 of its PCA in manchego-style cheeses. Similarly, the HCA analysis developed by these researchers allowed them to group the results based on ripening days.

In addition to the above, it is important to highlight that the variables of TPC, AA by DPPH, and AA by FRAP also significantly influenced the PC1 obtained in the present study (Figure 4a). This may be related to the high antioxidant capacity of curcumin added at different concentrations in the different cheeses evaluated. In addition, during maturation due to proteolysis, peptides are produced whose antioxidant activity influences the variables of AA by DPPH and FRAP.

On the other hand, PC2 is predominantly influenced by lipolysis (FFAs) and a* variables mainly related to cheeses made with bixin (Figure 4b). However, by increasing the concentration of NEC added to the cheeses, the influence on these variables decreases consistently throughout the different days of ripening (Figure 4b). The foregoing is consistent with what is described and discussed in Section 3.5 since it is appreciated that an increase in NEC concentration in cheeses causes less formation of free fatty acids than cheeses added with bixin (see Figure 2).

## 4. Conclusions

The influence of the addition of NEC to the milk on manchego-style cheese chemical composition, proteolysis, and lipolysis was assessed. Cheeses with the same moisture content, total protein, and water activity were obtained, although the addition of NEC significantly modified the proteolytic and lipolytic pattern of the cheese throughout the ripening period. Primary proteolysis of control and experimental cheeses was broadly similar. However, secondary proteolysis and lipolysis were different for control and experimental cheeses. The addition of NEC to the milk resulted in a significantly increased total phenolic content and antioxidant activity of the manchego-style cheese, which confirmed that NEC was possessed strong antioxidant activity to protect the cheese from oxidation, thereby improving the functionality of manchego-style cheese. Based on these results, NEC could promote the development of innovative functional dairy products with beneficial health effects.

## Figures and Tables

**Table 1:** Chemical composition of the manchego-style cheeses at different ripening times.

Cheese Samples	Ripening Time (Days)	Moisture (%)	Fat (%)	Total Protein (%)	Ash (%)	a_w_
B 10 ppm	0	46.66 ± 0.77 ^a^	27.20 ± 0.43 ^b^	22.09 ± 0.26 ^a^	3.40 ± 0.03 ^a^	0.973 ± 0.002
	20	46.40 ± 3.31 ^ab1^	26.72 ± 0.80 ^b^	22.32 ± 0.09 ^ab12^	3.53 ± 0.02 ^bc^	0.972 ± 0.001
	40	45.41 ± 1.56 ^ab^	23.41 ± 1.45 ^a^	22.24 ± 0.15 ^ab^	3.50 ± 0.02 ^b1^	0.972 ± 0.001
	60	44.89 ± 2.18 ^ab^	25.25 ± 0.79 ^ab1^	22.47 ± 0.43 ^ab^	3.60 ± 0.03 ^cd1^	0.970 ± 0.002
	80	43.48 ± 1.60 ^b^	26.82 ± 1.22 ^b1^	22.91 ± 0.41 ^b^	3.64 ± 0.03 ^d1^	0.970 ± 0.001
C 5 ppm	0	46.58 ± 2.36 ^a^	27.37 ± 0.55 ^bc^	22.20 ± 0.18	3.38 ± 0.11 ^a^	0.975 ± 0.001 ^a^
	20	46.61 ± 0.88 ^a1^	24.68 ± 1.25 ^ab^	22.41 ± 0.38 ^12^	3.58 ± 0.03 ^b^	0.973 ± 0.003 ^a^
	40	44.92 ± 2.18 ^ab^	24.29 ± 2.23 ^a^	22.54 ± 0.23	3.57 ± 0.05 ^b1^	0.973 ± 0.001 ^a^
	60	42.73 ± 1.85 ^b^	27.49 ± 1.47 ^bc12^	22.89 ± 0.41	3.62 ± 0.06 ^b1^	0.972 ± 0.003 ^ab^
	80	42.57 ± 2.69 ^b^	28.91 ± 2.47 ^c12^	22.73 ± 0.29	3.71 ± 0.01 ^c1^	0.969 ± 0.002 ^b^
C 7.5 ppm	0	45.70 ± 1.18 ^a^	26.89 ± 0.54 ^b^	22.17 ± 0.19 ^a^	3.45 ± 0.04 ^a^	0.974 ± 0.001 ^a^
	20	45.13 ± 0.94 ^a12^	26.01 ± 2.12 ^b^	21.89 ± 0.34 ^a1^	3.58 ± 0.02 ^b^	0.973 ± 0.003 ^ab^
	40	43.68 ± 2.80 ^ab^	25.09 ± 2.65 ^a^	22.77 ± 0.53 ^ab^	3.61 ± 0.07 ^b12^	0.973 ± 0.004 ^ab^
	60	42.69 ± 2.50 ^ab^	28.57 ± 0.61 ^c2^	23.34 ± 0.12 ^b^	3.60 ± 0.02 ^b1^	0.970 ± 0.001 ^b^
	80	41.13 ± 1.29 ^b^	27.63 ± 1.17 ^b12^	23.44 ± 0.37 ^b^	3.66 ± 0.03 ^b1^	0.971 ± 0.001 ^ab^
C 10 ppm	0	45.34 ± 1.95 ^a^	28.12 ± 1.22 ^b^	22.40 ± 0.61 ^a^	3.47 ± 0.09 ^a^	0.973 ± 0.001 ^a^
	20	42.42 ± 0.11 ^b2^	27.58 ± 2.31 ^b^	23.13 ± 0.37 ^ab2^	3.60 ± 0.03 ^b^	0.972 ± 0.000 ^a^
	40	42.44 ± 1.17 ^ab^	24.41 ± 0.72 ^a^	22.79 ± 0.63 ^ab^	3.72 ± 0.06 ^c2^	0.972 ± 0.003 ^ab^
	60	42.51 ± 0.44 ^b^	29.46 ± 2.00 ^b2^	23.42 ± 1.40 ^b^	3.71 ± 0.06 ^c2^	0.971 ± 0.002 ^ab^
	80	41.72 ± 1.63 ^b^	30.08 ± 1.31 ^b2^	23.55 ± 0.62 ^b^	3.85 ± 0.04 ^d2^	0.968 ± 0.003 ^b^

Results are expressed as mean (*n* = 3) ± standard deviation. ^a–d^ Means in each column with different letters were significantly affected by ripening time (*p* < 0.05); ^1,2^ means in each column with different numbers were significantly different between cheese samples at a similar ripening time (*p* < 0.05). a_w_ = water activity.

## Supplementary Materials

The following are available online at https://www.mdpi.com/article/10.3390/foods10071579/s1, Table S1: Proportion of saturated fatty acid (% SFA) in Manchego-style cheeses at different ripening times (Data from Figure 1a), Table S2: Proportion of monounsaturated fatty acid (% MUFA) in Manchego-style cheeses at different ripening times (Data from Figure 1b), Table S3: Proportion of polyunsaturated fatty acid (% PUFA) in Manchego-style cheeses at different ripening times (Data from Figure 1c), Table S4: Proportion of short-chain fatty acid (% SCFA) in Manchego-style cheeses at different ripening times (Data from Figure 1d), Table S5: Proportion of medium-chain fatty acid (% MCFA) in Manchego-style cheeses at different ripening times (Data from Figure 1e), Table S6: Proportion of long-chain fatty acid (% LCFA) in Manchego-style cheeses at different ripening times (Data from Figure 1f), Table S7: Lipolysis of Manchego-style cheeses ripened at 10 °C, expressed as free fatty acids (FFAs) content (meq KOH/100 g fat) B = bixin, C = curcumin (Data from Figure 2), Table S8: Proteolysis of Manchego-style cheeses ripened at 10 °C, expressed as % soluble nitrogen (SN) at pH 4.6 (pH 4.6-SN)/total nitrogen (TN) B = bixin, C = curcumin (Data from Figure 3a), Table S9: Proteolysis of Manchego-style cheeses ripened at 10 °C, expressed as % soluble nitrogen in trichloroacetic acid (TCA-SN)/TN. B = bixin, C = curcumin (Data from Figure 3b), Table S10: Proteolysis of Manchego-style cheeses ripened at 10 °C, expressed as % soluble nitrogen in phosphotungstic acid (PTA-SN)/TN. B = bixin, C = curcumin (Data from Figure 3c).
