# Supplementary material for: Ripening Changes of the Chemical Composition, Proteolysis, and Lipolysis of a Hair Sheep Milk Mexican Manchego-Style Cheese: Effect of Nano-Emulsified Curcumin
Source: Foods. 2021 Jul 7;10(7):1579. doi: 10.3390/foods10071579 (PMC8306841; doi:10.3390/foods10071579)
Supplement: Supplementary file 1 [file foods-10-01579-s001.zip › foods-1267957-supplementary.pdf]

SUPPLEMENTARY MATERIALS

Table S1. Proportion of saturated fatty acid (% SFA) in Manchego-style cheeses at different ripening times (Data from Figure 1a)

	Ripening times (days)				
	0	20	40	60	80
B 10 ppm	70.22±0.45 a A	70.19±2.02 a A	71.75±1.85 a A	71.70±2.85 ab A	71.76±3.05 a A
C 5 ppm	69.58±0.60 a B	73.74±1.40 a A	73.70±0.87 a A	73.73±1.43 a A	70.91±2.37 a AB
C 7.5 ppm	69.80±0.46 a A	75.42±2.09 a A	74.72±6.89 a A	72.26±0.24 ab A	71.81±1.53 a A
C 10 ppm	70.18±0.67 a A	74.70±2.39 a A	73.78±5.52 a A	69.07±0.36 b A	68.44±0.49 a A

Each entry represents the means ± standard deviation of three replicates. Entries bearing different lowercase letters in the same column denote significant differences ($p < 0.05$). Entries bearing different capital letters in the same row denote significant differences ($p < 0.05$).

Table S2. Proportion of monounsaturated fatty acid (% MUFA) in Manchego-style cheeses at different ripening times (Data from Figure 1b)

	Ripening times (days)				
	0	20	40	60	80
B 10 ppm	27.25±1.81 a A	25.53±2.67 a A	25.59±1.62 a A	25.66±2.56 a A	27.09±0.36 a A
C 5 ppm	27.81±0.59 a A	23.89±1.22 a B	23.90±1.23 a B	23.94±0.75 a B	26.48±2.23 a AB
C 7.5 ppm	27.57±0.45 a A	22.48±1.92 a A	22.91±6.13 a A	25.12±0.17 a A	25.65±1.48 a A
C 10 ppm	28.18±0.32 a A	23.08±2.10 a A	23.88±5.05 a A	27.14±0.58 a A	28.70±0.50 a A

Each entry represents the means ± standard deviation of three replicates. Entries bearing different lowercase letters in the same column denote significant differences ($p < 0.05$). Entries bearing different capital letters in the same row denote significant differences ($p < 0.05$).

Table S3. Proportion of polyunsaturated fatty acid (% PUFA) in Manchego-style cheeses at different ripening times (Data from Figure 1c)

	Ripening times (days)				
	0	20	40	60	80
B 10 ppm	2.69±0.11 a A	2.56±0.22 a A	2.64±0.30 a A	2.67±0.27 a A	2.71±0.38 a A
C 5 ppm	2.62±0.02 a A	2.36±0.13 a A	2.37±0.18 a A	2.34±0.18 a A	2.61±0.14 a A
C 7.5 ppm	2.63±0.06 a A	2.10±0.17 a A	2.37±0.77 a A	2.55±0.14 a A	2.62±0.07 a A
C 10 ppm	2.68±0.09 a A	2.22±0.29 a A	2.34±0.49 a A	2.75±0.03 a A	2.86±0.02 a A

Each entry represents the means ± standard deviation of three replicates. Entries bearing different lowercase letters in the same column denote significant differences ($p < 0.05$). Entries bearing different capital letters in the same row denote significant differences ($p < 0.05$).

Table S4. Proportion of short-chain fatty acid (% SCFA) in Manchego-style cheeses at different ripening times (Data from Figure 1d)

	Ripening times (days)				
	0	20	40	60	80
B 10 ppm	9.95±0.88 a A	15.86±1.43 a A	12.39±5.13 a A	12.98±4.23 bc A	12.51±4.43 a A
C 5 ppm	9.05±0.28 a B	20.41±1.93 a A	19.72±3.48 a A	20.44±1.34 a A	13.15±0.36 a B
C 7.5 ppm	9.26±1.04 a A	22.13±4.37 a A	18.54±12.22 a A	16.28±0.68 ab A	9.28±1.39 a A
C 10 ppm	10.02±0.69 a A	20.77±5.60 a A	16.14±10.18 a A	9.53±2.01 c A	8.27±0.61 a A

Each entry represents the means ± standard deviation of three replicates. Entries bearing different lowercase letters in the same column denote significant differences ($p < 0.05$). Entries bearing different capital letters in the same row denote significant differences ($p < 0.05$).

Table S5. Proportion of medium-chain fatty acid (% MCFA) in Manchego-style cheeses at different ripening times (Data from Figure 1e)

	Ripening times (days)				
	0	20	40	60	80
B 10 ppm	25.11±0.39 a A	23.38±1.65 a A	23.89±1.11 ab A	25.32±1.13 a A	22.25±3.28 a A
C 5 ppm	25.48±0.27 a A	21.14±1.30 a A	21.73±1.86 ab A	21.12±0.57 a A	22.88±3.15 a A
C 7.5 ppm	25.10±1.20 a AB	21.75±0.79 a AB	21.50±0.10 b B	25.06±3.20 a AB	26.85±2.45 a A
C 10 ppm	24.94 ±0.7 1 a AB	22.25±0.57 a B	24.77±0.84 a AB	24.05±0.60 a AB	25.64±1.89 a A

Each entry represents the means ± standard deviation of three replicates. Entries bearing different lowercase letters in the same column denote significant differences ($p < 0.05$). Entries bearing different capital letters in the same row denote significant differences ($p < 0.05$).

Table S6. Proportion of long-chain fatty acid (% LCFA) in Manchego-style cheeses at different ripening times (Data from Figure 1f)

	Ripening times (days)				
	0	20	40	60	80
B 10 ppm	64.94±0.94 a A	64.23±3.52 a A	63.14±4.05 a A	62.17±4.62 a A	61.88±4.48 a A
C 5 ppm	65.47±0.29 a A	58.44±3.13 a A	58.55±2.14 a A	58.44±3.09 a A	63.98±5.80 a A
C 7.5 ppm	65.64±0.64 a A	55.12±4.76 a A	56.39±14.57 a A	62.21±0.58 a A	63.87±3.27 a A
C 10 ppm	65.04±1.34 a A	56.97±5.51 a A	58.22±11.98 a A	65.70±1.21 a A	66.68±0.67 a A

Each entry represents the means ± standard deviation of three replicates. Entries bearing different lowercase letters in the same column denote significant differences ($p < 0.05$). Entries bearing different capital letters in the same row denote significant differences ($p < 0.05$).

Table S7. Lipolysis of Manchego-style cheeses ripened at 10 ºC, expressed as free fatty acids (FFAs) content (meq KOH/100 g fat) B = bixin, C = curcumin (Data from Figure 2)

	Ripening times (days)				
	0	20	40	60	80
B 10 ppm	1.84±0.03 a D	2.98±0.16 a BC	3.76±0.16 a A	3.13±0.25 a B	2.70±0.08 a C
C 5 ppm	1.80±0.43 a B	2.47±0.29 ab AB	3.04±0.13 b A	2.14±0.14 b B	1.96±0.17 b B
C 7.5 ppm	1.71±0.20 a C	2.25±0.11 bc AB	2.46±0.08 c A	1.97±0.13 b BC	1.29±0.14 c D
C 10 ppm	1.61±0.15 a B	1.88±0.19 c AB	2.21±0.08 c A	1.99±0.23 b AB	1.78±0.07 b B

Each entry represents the means ± standard deviation of three replicates. Entries bearing different lowercase letters in the same column denote significant differences ($p < 0.05$). Entries bearing different capital letters in the same row denote significant differences ($p < 0.05$).

Table S8. Proteolysis of Manchego-style cheeses ripened at 10 ºC, expressed as % soluble nitrogen (SN) at pH 4.6 (pH 4.6-SN)/total nitrogen (TN) B = bixin, C = curcumin (Data from Figure 3a)

	Ripening times (days)				
	0	20	40	60	80
B 10 ppm	7.88±0.58 a C	13.59±1.24 a B	17.90±2.26 a AB	17.12±1.87 a AB	20.07±1.98 a A
C 5 ppm	8.92±0.32 a D	13.80±0.81 a C	16.37±0.29 a B	17.88±1.46 a B	22.61±0.75 a A
C 7.5 ppm	8.55±0.13 a D	13.47±0.71 a C	17.37±0.64 a B	19.43±0.81 a AB	21.85±1.60 a A
C 10 ppm	8.63±0.89 a D	13.48±0.21 a C	17.72±0.96 a B	20.31±2.19 a AB	22.37±0.67 a A

Each entry represents the means ± standard deviation of three replicates. Entries bearing different lowercase letters in the same column denote significant differences ($p < 0.05$). Entries bearing different capital letters in the same row denote significant differences ($p < 0.05$).

Table S9. Proteolysis of Manchego-style cheeses ripened at 10 ºC, expressed as % soluble nitrogen in trichloroacetic acid (TCA-SN)/TN. B = bixin, C = curcumin (Data from Figure 3b)

	Ripening times (days)				
	0	20	40	60	80
B 10 ppm	6.05±0.19 a D	11.05±0.40 a C	13.23±1.53 a BC	14.68±0.84 a AB	16.43±1.13 a A
C 5 ppm	6.36±0.51 a E	10.61±0.30 a D	12.70±0.34 a C	14.02±0.47 a B	16.71±0.67 a A
C 7.5 ppm	6.03±0.64 a D	11.04±0.63 a C	13.31±0.94 a BC	15.73±0.70 a AB	16.24±1.54 a A
C 10 ppm	6.31±0.39 a D	11.09±0.48 a C	14.76±1.57 a B	15.08±0.86 a B	17.55±0.59 a A

Each entry represents the means ± standard deviation of three replicates. Entries bearing different lowercase letters in the same column denote significant differences ($p < 0.05$). Entries bearing different capital letters in the same row denote significant differences ($p < 0.05$).

Table S10. Proteolysis of Manchego-style cheeses ripened at 10 ºC, expressed as % soluble nitrogen in phosphotungstic acid (PTA-SN)/TN. B = bixin, C = curcumin (Data from Figure 3c)

	Ripening times (days)				
	0	20	40	60	80
B 10 ppm	4.05±0.49 a AB	4.23±0.58 b AB	3.81±0.21 b B	4.38±0.02 c AB	4.93±0.06 b A
C 5 ppm	3.95±0.27 a C	4.66±0.63 ab BC	6.27±0.22 a A	5.31±0.41 bc AB	5.51±0.07 b AB
C 7.5 ppm	4.59±0.37 a C	4.99±0.15 ab BC	6.79±0.73 a A	6.03±0.53 b AB	6.88±0.43 a A
C 10 ppm	4.14±0.24 a D	5.61±0.43 a C	7.04±0.19 a B	8.25±0.32 a A	7.38±0.44 a AB

Each entry represents the means ± standard deviation of three replicates. Entries bearing different lowercase letters in the same column denote significant differences ($p < 0.05$). Entries bearing different capital letters in the same row denote significant differences ($p < 0.05$).
